# 

**Published:** 1918-01-26

**Authors:** 


Rates of Subscription: Twelve months, 6/6; Six months, 4/-; three months, 2/-, post free to any address in the United Kingdom. Abroad, Twelve months, 8/8; Six months, 5/-; Three months, 2/6, post free. **THE HOSPITAL "Index" to Volume LXII., April 1917 to September 1917, is now ready, price 2½d. per copy, post free.** Address The Manager, The Hospital, "The Hospital" Building, 28/29 Southampton Street, Strand, London, W.C. 2.

West Ham Education Committee has had under consideration the scale of remuneration for school nurses, which is at present £70, rising by annual increments of £10 to £100 a year. It is proposed to rescind this scale and fix the salary at £80, rising to £100.

### Matrons' and Sisters' Appointments.

AUXILIARY HOSPITAL, HOLMFIRTH, HUDDERSFIELD.—Sister Costella has been appointed night sister. She was trained at Newark-on-Trent Hospital, and has been theatre sister at Doncaster Infirmary and staff nurse at Mexborough Military Hospital.

CAMERON HOSPITAL, WEST HARTLEPOOL.—Miss Agnes Brown has been appointed sister. She was trained at the General Hospital, Stroud, where she has since been sister.

COUNTY HOSPITAL, YORK.—Miss Margaret K. Steele has been appointed lady superintendent of nurses. She was trained at St. Bartholomew's Hospital, London, and has since held the posts of night sister at the Royal Hospital for Sick Children, Edinburgh, housekeeping sister at the London Homœopathic Hospital, and home sister and assistant matron at St. Bartholomew's Hospital, Rochester. Miss Elsie Hughes has been appointed night superintendent. She was trained at Wolverhampton General Hospital, where she was later ward sister, and she has since been senior sister at the Ear and Throat Hospital, Birmingham.

DOWNS SANATORIUM, SUTTON, SURREY.—Miss Gertrude Fisher has been appointed sister. She was trained at the General Hospital, Wolverhampton, and has been sister at the Royal Sea Bathing Hospital, Margate, and at the Baguley Sanatorium, Manchester.

HIGHBURY AUXILIARY HOSPITAL, MOSELEY, BIRMINGHAM.—Miss Alice A. Warriner has been appointed night sister. She was trained at the Grimsby and District Hospital and at the London Temperance Hospital, has been sister at the Royal Victoria Hospital, Dover, the General Hospital, Wolverhampton, the General Hospital, Birmingham, and matron of Corbett Hospital, Stourbridge.

NORTH DEVON INFIRMARY, BARNSTAPLE.—Miss L. E. Moss has been appointed matron. She was trained at the Derbyshire Royal Infirmary, later being sister there.

VICTORIA HOSPITAL FOR SICK CHILDREN, HULL.—Miss Florence Cartwright has been appointed sister of the out-patient department and masseuse. She was trained at the Royal Infirmary, Glasgow, and in massage at the Bristol Royal Infirmary. She has been massage sister and second superintendent of nurses at Brownlow Hill Infirmary, Liverpool, and has seen foreign service in the Balkans.

WEST HOUSE ROYAL ASYLUM, EDINBURGH.—Miss Lilian Arrowsmith has been appointed deputy-matron. She was trained at Ancoats Hospital, Manchester, has been medical, sanatorium, and night sister at Dumfries Royal Infirmary, sister at Leith Hospital, assistant matron at West House Morningside Asylum, and matron of Exeter City Asylum.

**NOTE.—Particulars of Appointments for insertion in this column will be welcomed.**

### The Book Market.

LIST OF NEW BOOKS RECEIVED FROM THE FOLLOWING PUBLISHERS :—

Baillière, Tindall and Cox : " Chemistry for Beginners." By C. T. Kingzett, F.I.C., F.C.S. 2s. 6d. net.

John Bale, Sons and Danielson, Ltd. : ' The Discharged Soldier : His Treatment in Relation to the Treatment of Consumption as a Whole." By Thomas Denver. Price 1s.

J. B. Lippincott Co. ; " The Battle with Tuberculosis and How to Win it." By D. Macdougall King, M.B. 6s. net.

Longmans, Green and Co. : " The Control of the Drink Trade." By Henry Carter. 7s. 6d. net.

**Papers, Pamphlets, etc.**

John Wright and Sons, Ltd. : " The British Journal of Surgery. October 1917." 8s. 6d. net.

### Church Collections for the Hospital Sunday Fund.

THE following is an analysis prepared by the *National Church* of the collections in various places of worship in 1917 for the Metropolitan Hospital Sunday Fund : Church of England, £28,133 13s. 6d. ; Jews, £1,766 12s. 11d. ; Congregationalists, £1,563 3s. 11d. ; Presbyterians, £1,114 18s. 10d. ; Wesleyans, £984 0s. 5d. ; Roman Catholics, £833 3s. 9d. ; Baptists, £831 1s. ; Church of Scotland, £450 16s. 2d. ; Unitarians, £411 5s. 9d. ; Methodist (Primitive), £174 ; Greek Church, £167 1s. 3d. ; Catholic Apostolic, £88 11s. 3d. ; Methodists (Welsh Calvinistic), £57 3s. 1d. ; Foreign Protestants, £51 15s. ; Society of Friends, £46 9s. 4d. ; Reformed Episcopal Church, £42 19s. 4d. ; German Lutherans, £19 9s. ; Methodist (United), £18 8s. 6d. ; Free Church, £12 10s. ; Moravians, £6 5s. 4d. ; Swedenborgians, £1 15s. ; Various, £525 14s. 2d.

